# The changing face of thyroid cancer in a population-based cohort

**DOI:** 10.1002/cam4.103

**Published:** 2013-06-26

**Authors:** K Alok Pathak, William D Leslie, Thomas C Klonisch, Richard W Nason

**Affiliations:** 1 Head and Neck Surgical Oncology, Cancer Care ManitobaWinnipeg, Canada; 2 Nuclear Medicine, Cancer Care ManitobaWinnipeg, Canada; 3 Human Anatomy and Cell Science, University of ManitobaWinnipeg, Canada

**Keywords:** Anaplastic, epidemiology, incidence, outcome, survival, trend

## Abstract

In North America, the incidence of thyroid cancer is increasing by over 6% per year. We studied the trends and factors influencing thyroid cancer incidence, its clinical presentation, and treatment outcome during 1970–2010 in a population-based cohort of 2306 consecutive thyroid cancers in Canada, that was followed up for a median period of 10.5 years. Disease-specific survival (DSS) and disease-free survival were estimated by the Kaplan–Meier method and the independent influence of various prognostic factors was evaluated by Cox proportional hazard models. Cumulative incidence of deaths resulting from thyroid cancer was calculated by competing risk analysis. A *P*-value <0.05 was considered to indicate statistical significance. The age standardized incidence of thyroid cancer by direct method increased from 2.52/100,000 (1970) to 9.37/100,000 (2010). Age at diagnosis, gender distribution, tumor size, and initial tumor stage did not change significantly during this period. The proportion of papillary thyroid cancers increased significantly (*P *< 0.001) from 58% (1970–1980) to 85.9% (2000–2010) while that of anaplastic cancer fell from 5.7% to 2.1% (*P* < 0.001). Ten-year DSS improved from 85.4% to 95.6%, and was adversely influenced by anaplastic histology (hazard ratio [HR] = 8.7; *P* < 0.001), male gender (HR = 1.8; *P *= 0.001), TNM stage IV (HR = 8.4; *P* = 0.001), incomplete surgical resection (HR = 2.4; *P* = 0.002), and age at diagnosis (HR = 1.05 per year; *P* < 0.001). There was a 373% increase in the incidence of thyroid cancer in Manitoba with a marked improvement in the thyroid cancer-specific survival that was independent of changes in patient demographics, tumor stage, or treatment practices, and is largely attributed to the declining proportion of anaplastic thyroid cancers.

This article shows there is an increase in the incidence of thyroid cancers of all sizes in a population cohort in Canada. The improvement in thyroid cancer survival is due to reduced proportion of anaplastic thyroid cancer.

## Introduction

Thyroid cancer is the most common malignant endocrine tumor and is the seventh most common cancer seen in Canadians with an estimated 5650 new thyroid cancers diagnosed in 2012 1. In Canada, the incidence of thyroid cancer is increasing more rapidly than any other cancer; by 6.8% per year in Canadian males (1998–2007) and by 6.9% per year in Canadian females (2002–2007) 1. Similar trends have been reported globally 2–13. By direct method, the age standardized incidence rate (ASIR) of thyroid cancer per 100,000 Canadians has increased from 1.1 in 1970–1972 to 6.1 in 2012 for males, and from 3.3 to 22.2 in females during the same period 1–14. The trends in the United States (US) mirror that of Canada with a threefold increase in the incidence of thyroid cancer from 4.85/100,000 in 1975 to 14.25/100,000 in 2009 and an annual percent increase (2000–2009) of 6.0% for the US males and 6.9% for the US females 15. The age and delay adjusted incidence rate of thyroid cancer between 2006 and 2010 was 6.1/100,000 for the US males and 18.2/100,000 for the US females using joint point regression program. An estimated 56,460 new cases of thyroid cancers are likely to be seen in the US in 2012 and 1780 will die from it. The life time probability of developing a thyroid cancer for a Canadian female is 1 in 71 (1.4%) but only 1 in 1374 (0.1%) will actually die from it 16. Canadian males have a lower life time risk of developing thyroid cancer at 1 in 223 (0.4%) with the risk of death from thyroid cancer at 1 in 1937 (0.1%).

Although the incidence of thyroid cancer has been rising, it had an excellent 5-year relative survival ratio of 98% in 2011 16. Thyroid cancers represent a conglomerate of different histological types that have diverse clinical behavior. Differentiated thyroid cancers (papillary and follicular) typically have an excellent survival, whereas poorly differentiated and anaplastic thyroid cancers have a very poor outcome. Although the time trends in the incidence of thyroid cancer in Canada have been reported earlier 14–17, little has been recorded about the trends in outcome of thyroid cancer in a population cohort. The objective of this study was to assess the trends in the incidence, clinical presentation, treatment practices, and disease outcome of thyroid cancer in a population-based cohort spanning four decades from 1970 to 2010.

## Patients and Methods

### Study group

Manitoba thyroid cancer cohort is a historical cohort that includes all 2306 consecutive thyroid cancers observed in 2296 patients in the province of Manitoba, Canada, from 1 January 1970 to 31 December 2010, as registered in the Manitoba Cancer Registry. The Manitoba Cancer Registry is a member of the North American Association of Central Cancer Registries which administers a program that reviews member registries for their ability to produce complete, accurate, and timely data. CancerCare Manitoba is the sole comprehensive cancer care center in the province where all the cancer patients from the province are primarily referred and the Manitoba Cancer Registry is a part of CancerCare Manitoba. Ethics approval for this study was obtained from the Research Ethics Board at the University of Manitoba.

We reviewed individual electronic and paper records of diagnosis and treatment for this cohort of patient. The primary source of diagnostic information included 2148 pathology reports, 80 discharge summaries, 56 autopsy records, 18 operative reports, and 4 death certificates. ASIR was calculated by direct method (1). Age-specific incidence rates were initially calculated by using the age distributions of the newly diagnosed thyroid cancers cases in the province and that of the provincial population for each year obtained from the Manitoba Health's population registry, Manitoba Bureau of Statistics 18 and Statistics Canada 19. Age-specific incidences were then applied to the 1991 standard Canadian population 1 to calculate the ASIR per 100,000 population.

Patient demographics, extent of disease at initial presentation; the treatment modalities employed; pathology details; cancer recurrences during follow-up and final outcome status as of 1 January 2013 were recorded. All patients who migrated out of province (considered lost to follow-up) or died during the study period, were censored at that point in time. All cases were restaged according to the American Joint Cancer Committee/Union Internationale Contre le Cancer (AJCC/UICC) staging for thyroid cancer (7th edition, 2009); the topography and the histology were recoded by WHO International Classification of Diseases for Oncology (3rd Edition) codes and the disease, signs, and symptoms by International Classification of Diseases (10th edition) codes to ensure uniformity. The pathology and treatment details of 683 (29.6%) were independently reviewed for accuracy as a part of a collaborative staging project.

### Statistical analysis

Analysis of variance was used to compare group means, and categorical data were compared using the Pearson chi-square test with continuity correction, as appropriate. A *P*-value <0.05 was considered to indicate statistical significance and 95% confidence intervals were used to express reliability in the estimates. After checking for normality assumption the mean and standard deviation were used to express normally distributed data (such as age of the patients) and median with interquartile range were used for nonnormally distributed data (such as tumor size and follow-up). The data were managed and analyzed using SPSS for Windows version 20.0 (SPSS Inc., Chicago, IL). Disease-free survival (DFS) and disease-specific survival (DSS) were estimated by the Kaplan–Meier product limit method, and the effect of individual prognostic factors on survival was assessed by using the log rank test. Multivariate analyses were performed with Cox proportional hazard models to assess the independent effect of prognostic factors on DSS after testing for the proportional hazard assumption. The competing influence of other causes of mortality, such as death due to a second primary tumor or noncancer deaths, was analyzed by competing risk regression model using STATA version 12 (StataCorp. TX).

## Results

### Study cohort

**Figure 1:**
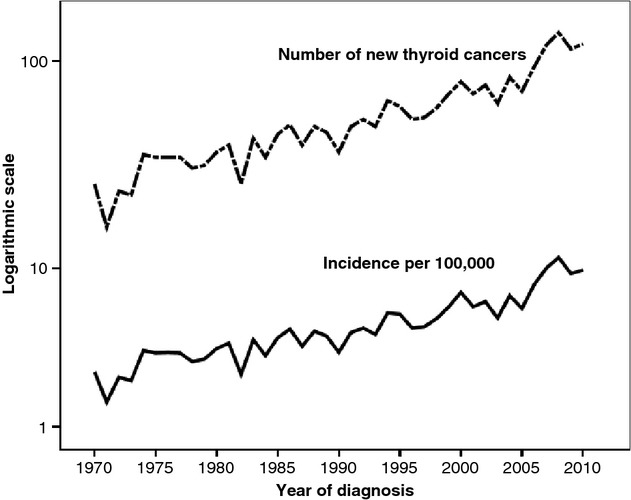
Trends in the age standardized incidence, the number of newly diagnosed thyroid cancers in Manitoba, Canada (1970–2010).

In total, 2306 thyroid cancers were observed in Manitoba in 2296 patients from 1970 to 2010. Nine patients had a synchronous second primary tumor of a different histology along with papillary thyroid cancer (follicular-3, hürthle cell-3, medullary-2, poorly differentiated-1), while one patient had a metachronous second papillary carcinoma in the contra-lateral thyroid lobe 25 years after initial management. According to 2011 census, the population of Manitoba was recorded at 1,208,268 that increased by an average of 0.56% per year from 1970 level of 988,245. The number of newly diagnosed thyroid cancers increased from 22 in 1970 to 122 in 2010, and the ASIR per 100,000 for thyroid cancer from 2.52 (95% confidence interval [CI] = 1.57–3.83) in 1970 to 9.37 (95% CI = 7.65–11.08) in 2010 (Fig. 1). The ASIR per 100,000 rose from 0.72 (95% CI = 1.57–3.83) in 1970 to 4.94 (95% CI = 3.35–7.03) in 2010 for males and the respective rates for females went up from 4.28 (95% CI = 2.56–6.71) to 13.75 (95% CI = 10.96–17.04). The ASIR per 100,000 for anaplastic thyroid cancers fell from 0.11 (95% CI = 0.05–0.19) during 1970–1980 to 0.05 (95% CI = 0.02–0.11) in 2001–2010 for both sexes and the respective rates for papillary thyroid cancer went up from 0.93 (95% CI = 0.75–1.15) to 6.64 (95% CI = 6.17–7.11).

The Manitoba thyroid cancer cohort included 570 (24.8%) males and 1726 (75.2%) females with a mean age (±standard deviation) of 49 ± 18 years. The mean age of patients at the time of diagnosis, median tumor size, and the tumor stage at presentation showed statistically nonsignificant change (*P* > 0.05, NS) over the past four decades (Table 1). Thyroid cancers ≤10 mm (microcarcinoma) represented 23.4% of all thyroid cancers and this proportion did not change significantly (*P* = 0.87) during the study period. Lymph node involvement was present in 23.7% cases and distant metastasis in 3.9% cases at the time of diagnosis. Multifocal disease was observed in 32.3% cases, gross extrathyroidal extension of tumor in 20.9% cases, and complete surgical excision of gross tumor was achieved in 96.1% cases. The distribution of T stage (*P* = 0.50), N stage (*P* = 0.27), and M stage (*P* = 0.61) as well as the proportion of patients with multifocal disease (*P* = 0.26), gross extrathyroidal extension of tumor (*P* = 0.66) or with complete excision of tumor (*P *= 0.40) remained unchanged during the study period.

**Table:** Clinical characteristics of thyroid cancer by decade of presentation

	1970–1980 (*N* = 331)	1981–1990 (*N* = 410)	1991–2000 (*N* = 594)	2001–2010 (*N* = 971)	*P*-value
Mean age in years	49.3 ± 18.4	47.5 ± 18.6	48.0 ± 18.7	49.1 ± 17.0	0.14 (NS)
Gender ratio (female:male)	2.5:1	2.8:1	3.6:1	3:1	0.45 (NS)
Median tumor size (interquartile range)	19.9 (15–22) mm	20 (20–25) mm	21 (20–24) mm	20 (19–22) mm	0.44 (NS)
Tumor size distribution					
≤1 cm	75 (22.6%)	117 (28.5%)	153 (25.8%)	242 (24.9%)	0.13 (NS)
1–2 cm	110 (33.2%)	93 (22.7%)	143 (24.1%)	247 (25.5%)	
2–4 cm	108 (32.6%)	152 (37.0%)	197 (33.1%)	310 (31.9%)	
>4 cm	38 (11.6%)	48 (11.8%)	101 (17.0%)	172 (17.7%)	
Stage I	207 (62.6%)	280 (68.4%)	380 (64.0%)	610 (62.8%)	0.22 (NS)
Stage II	23 (6.9%)	34 (8.3%)	62 (10.4%)	120 (12.4%)	
Stage III	32 (9.7%)	52 (12.6%)	62 (10.4%)	125 (12.9%)	
Stage IV	69 (20.8%)	44 (10.7%)	90 (15.2%)	116 (11.9%)	
Total thyroidectomy	94 (28.4%)	159 (38.9%)	293 (49.3%)	691 (71.2%)	<0.001
Adjuvant radioactive iodine	36 (10.9%)	79 (19.3%)	177 (29.8%)	603 (62.1%)	0.004
Histology					
Papillary (%)	192 (58.0%)	278 (67.8%)	459 (77.3%)	834 (85.9%)	<0.001
Follicular (%)	86 (26.0%)	70 (17.1%)	62 (10.4%)	50 (5.1%)	
Hürthle cell (%)	3 (0.9%)	24 (5.9%)	22 (3.7%)	36 (3.7%)	
Poorly differentiated (%)	9 (2.7%)	1 (0.2%)	3 (0.5%)	3 (0.3%)	
Medullary (%)	17 (5.1%)	24 (5.9%)	17 (2.9%)	21 (2.2%)	
Anaplastic (%)	19 (5.7%)	9 (2.2%)	24 (4.0%)	20 (2.1%)	
Unspecified (%)	5 (1.5%)	4 (1%)	7 (1.2%)	7 (0.7%)	
Median follow-up in months (95% CI)	419.4 (409.9–428.9)	304.9 (296.2–313.7)	188.4 (182.5–194.3)	68.5 (65.4–71.6)	<0.001
10 year DSS (*N* = 2296)	85.4 (82.9-90.0)%	92.2 (89.1–94.5)%	89.3 (86.5–91.6)%	95.6 (90.5–96.6)%	<0.001
Posttreatment 10 year DFS (*N* = 2065)	86.6 (81.1–90.6)%	88.9 (85.1–91.8)%	88.1 (84.7–90.6)%	91.9 (89.2–94.1)%	0.18 (NS)

Papillary thyroid cancer was the most common histological type, and the proportion of papillary thyroid cancers steadily increased from 58.0% (1970–1980) to 85.9% (2000–2010). Most of the thyroid cancers were classical papillary thyroid cancers (46.9%), followed by follicular (24.2%), encapsulated (2.3%), and microinvasive (1.6%) variants. Sclerosing, solid, tall cell, and columnar cell variants together accounted for only 1.9% of all thyroid cancers. During the same period, the proportion of all thyroid cancers that were diagnosed as follicular variants of papillary thyroid cancer increased from 12.4% to 32.9% (Table 1), whereas the proportion of follicular carcinoma fell from 26% to 5.1% (*P* < 0.001) and anaplastic carcinoma from 5.7% to 2.1% (*P* < 0.001). The proportion of medullary thyroid carcinoma has remained nearly unchanged. Total thyroidectomy was performed in 55.1% cases and 34.8% had adjuvant radioactive iodine (RAI). The percentage of the patients undergoing total thyroidectomy increased by two and a half times between 1970 and 1980 and 2001–2010 (*P* < 0.001) while those receiving adjuvant RAI increased by over five times (*P* = 0.004).

### Oncological outcome of thyroid cancer

**Figure 2:**
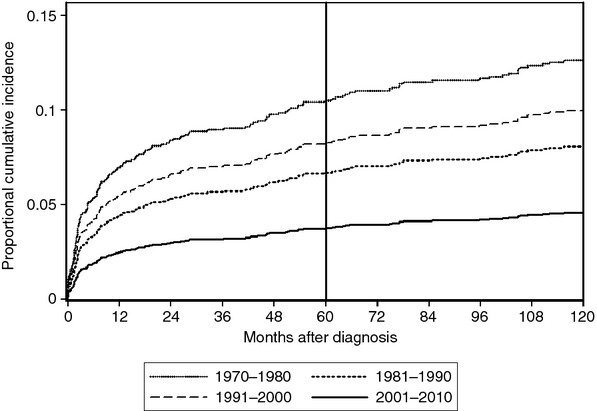
Cumulative incidence of death resulting from thyroid cancer over 10 years.

In all, 201 patients died from thyroid cancer (case fatality rate 8.8%). The case fatality rate for different histological types of thyroid cancer is summarized in Table 2. Of all deaths due to thyroid cancer, 63 (31.3%) had papillary carcinoma, 60 (29.9%) anaplastic, 24 (11.9%) follicular, 21 (10.4%) medullary, 15 (7.5%) hürthle cell, 8 (4%) poorly differentiated, and 10 (5%) had unspecified thyroid carcinoma. The 10-year DSS of Manitoba thyroid cancer cohort (*N* = 2296) was 91.8% (95% CI = 90.5–92.9%), which improved significantly from 85.4% (1970–1980) to 95.6% (2001–2010) (Table 1). Tumor histology had a very significant impact on the DSS and DFS survivals (Table 2). Papillary thyroid cancer had the best DSS rates (96.8% at 10 years and 94.6% at 20 years), whereas only 7.3% with anaplastic cancer survived for 10 years. The cumulative incidence function (CIF) by competing risk regression analysis (Fig. 2) shows an 8.1% reduction (from 12.6% to 4.5%) in death due to thyroid cancer at 10 years of follow-up.

**Table:** Case fatality rate and survival by histological types of thyroid cancers

	Case fatality rate	10 year DSS (95% CI)	20 year DSS (95% CI)	10 year DFS (95% CI)	20 year DFS (95% CI)
Papillary	3.6%	96.8 (95.7–97.7)%	94.6 (92.9–95.9)%	87.7 (85.9–89.4)%	84.8 (82.5–86.9)%
Follicular	9.0%	91.5 (87.0–94.5)%	88.6 (83.1–92.4)%	87.3 (82.4–91.0)%	86.1 (80.8–90.0)%
Hürthle cell	17.6%	81.4 (69.7–89.0)%	75.6 (61.2–85.2)%	69.8 (57.9–79.0)%	69.8 (57.9–79.0)%
Poorly differentiated	31.2%	74.1 (28.9–93.0)%	74.1 (28.9–93.0)%	27.3 (6.5–53.9)%	27.3 (6.5–53.9)%
Medullary	26.9%	77.6 (65.4–85.9)%	62.6 (47.5–74.5)%	52.0 (38.5–63.9)%	43.1 (28.9–56.6)%
Anaplastic	83.3%	7.3 (2.4–15.9)%	7.3 (2.4–15.9)%	1.4 (0.1–6.7)%	1.4 (0.1–6.7)%

Treatment outcomes were assessed in the 2065 patients who underwent primary surgical treatment with radical intent in Manitoba; after excluding cases that were diagnosed only on autopsy or death report (*n* = 60), those who died before surgery (*n* = 64), nonsurgical candidates (*n* = 35) or those who refused treatment (*n* = 24), and if primary surgery or follow-up performed in another province/country (*n* = 48). During median follow-up of 10.5 years (interquartile range = 4.8–18.8 years), 78 (3.8%) patients had posttreatment residual disease and another 200 (9.7%) patients had recurrence of disease at least 6 months after successful initial treatment. The recurrences were in the residual thyroid lobe or thyroid bed in 23 (11.5%) cases, in the central compartment of neck in 64 (32%), in the lateral compartment of neck in 44 (22%), at a distant metastatic site in 54 (27%), and at multiple sites in 15 (7.5%) cases. Ten-year DFS of the surgically treated patients was 89.3% (95% CI = 87.7–90.8%) that has not changed significantly over the last four decades (Table 1).

On multivariate analysis by Cox proportional hazard model, DSS was adversely influenced independently by nonpapillary histology especially anaplastic (hazard ratio [HR] = 8.7; 95% CI = 5.2–14.5; *P* < 0.001), male gender (HR = 1.8; 95% CI = 1.3–2.5; *P* = 0.001), TNM stage III (HR = 2.30; 95% CI = 1.00–5.29, *P* = 0.05), and IV (HR = 8.37; 3.99–17.54, *P* < 0.001), incomplete surgical resection (HR = 2.4; 95% CI = 1.4–4.2, *P* = 0.002), and age at diagnosis (HR = 1.05 per year; 95% CI = 1.03–1.06; *P* < 0.001). Each of these factors, except the histology has remained unchanged during the study period (Table 1). The extent of thyroidectomy, use of adjuvant RAI, or the decade of diagnosis did not have any significant influence on DSS (Table 3). Competing risk subhazard model confirmed these observations of Cox regression analysis.

**Table:** Multivariate analysis by Cox proportional hazard models for independent influence of prognostic factors on disease-specific survival

Prognostic factor	Hazard ratio (95% confidence interval)
Age at the time of diagnosis (per year)	1.05 (1.03–1.06), *P *< 0.001
Gender (male vs. female)	1.79 (1.28–2.51), *P *= 0.001
Extent of thyroidectomy (hemi vs. total)	0.991 (0.60–1.64), *P *= 0.971 (NS)
Completeness of resection (incomplete vs. complete)	2.40 (1.37–4.19), *P *= 0.002
Adjuvant radioactive iodine (RAI vs. no RAI)	1.59 (0.91–2.79), *P* = 0.101(NS)
AJCC TNM stage grouping	
Stage I	1.00 (reference)
Stage II	1.94 (0.78–4.84), *P* = 0.155 (NS)
Stage III	2.30 (1.00–5.29), *P* = 0.05
Stage IV	8.37 (3.99–17.54), *P *< 0.001
Histology	
Papillary	1.00 (reference)
Anaplastic	8.66 (5.18–14.49), *P *< 0.001
Medullary	2.62 (1.48–4.66), *P *= 0.001
Hürthle	2.23 (1.21–4.14), *P *= 0.010
Follicular	1.76 (1.01–3.09), *P* = 0.047
Decade of diagnosis	
1970–1980	1.00 (reference)
1981–1990	0.92 (0.56–1.53), *P *= 0.753 (NS)
1991–2000	1.40 (0.91–2.17), *P* = 0.126 (NS)
2001–2010	0.65 (0.40–1.08), *P* = 0.096 (NS)

## Discussion

A cohort is a population group, or subset thereof, that is followed over a period of time. The members of the cohort, based on defined criteria, share common experience which in this study was diagnosis of thyroid cancer in the province of Manitoba. By virtue of the residence in the same province our study cohort was expected to share similar risk of exposure and get similar standard of medical care in the publicly funded health care system of the province. The incidence trends of thyroid cancers in the US were reported by using the Surveillance, Epidemiology, and End Results (SEER) database, which is robust but it has its inherent limitations in terms of coverage, coding reliability, patient migration to an area that is not covered by SEER and limited information on the treatment details of the patients. These limitations make it difficult to interpret the time trend in treatment of thyroid cancer and its influence on thyroid cancer related survival. Due to the excellent treatment outcome of most thyroid cancers, randomized controlled trials to assess treatment response will require prohibitively large sample size and resources and therefore, they are not readily feasible 20. A population-based cohort with low attrition rates such as ours (only 2.1% loss to follow-up over 40 years) is the probably the best possible model to study the time trends in thyroid cancer survival. As all members of the cohort received the same treatment, that was the standard of care for their era, and their medical records were individually reviewed for accuracy and completeness; our study cohort was a reliable and stable model to study the trends and factors influencing survival of patients with thyroid cancer.

Our study shows that the incidence of thyroid cancer in the province of Manitoba has increased by 373% from 1970 to 2010 that has resulted largely from an increase in the number of papillary thyroid cancers (Table 1). The proportion of cancers classified as follicular variant of papillary thyroid cancer has almost tripled from 12.4% to 32.9%; this has also contributed to the increase in the proportion of papillary thyroid cancer. At the same time, the proportion of follicular thyroid cancer has decreased from 26% to 5.1%, possibly due to a change in the criteria for diagnosing papillary carcinoma after 1988, whereby, all lesions with typical cytological features (ground-glass nuclei, nuclear grooves, and psammoma bodies) were classified as papillary carcinoma even if they did not show papillae 21. Thus, many erstwhile follicular thyroid cancers are rechristened as follicular variant of papillary thyroid cancer. During the last four decades, the 10-year DSS has improved from 85.4% to 95.6%, whereas the 10 year DFS has remained stable (86.6–91.9%).

In 2012, thyroid cancers (*N* = 5650) were expected to account for 2.1% of all cancers (*N* = 267,700) in Canada 1. The number of the newly diagnosed thyroid cancer in the province of Manitoba has increased by over threefold in the last four decades which is congruent with reports from Canada and other countries 2–13. However, this increase in our study cohort has not been restricted to the smaller thyroid cancers as has been thought of earlier 2–17. We observed an increase in the number of thyroid cancers of all sizes, which is a true increase in the incidence of thyroid cancer in Manitoba and it cannot be explained only by over diagnosis of subclinical disease. A single cause for this increase is not apparent and this may be multifactorial due to iodine deficiency 22, radiation exposure 23,24, long-standing goiters, and family history 26. The shift in histological pattern of thyroid cancer has been reported from other regions also 27. The decrease in proportion of anaplastic carcinoma could be a result of more aggressive treatment of goiters and differentiated thyroid cancers by thyroidectomies before they could undergo anaplastic transformation. The proportion of tumors >2 cm in our cohort is higher than that reported earlier 7,16, because of the strict criteria for fine needle biopsy of thyroid in Manitoba. Most of the microcarcinomas in our study were incidentally detected in thyroidectomy or autopsy specimens. Even before the introduction of the American Thyroid Association guidelines 29, which recommend threshold sizes of >1 cm (hypoechoic solid nodule), 1–1.5 cm (iso or hyperechoic solid nodule), 1.5–2 cm (mixed cystic–solid nodule with suspicious features), and ≥2.0 cm (mixed cystic–solid nodule without suspicious features) in a low-risk patient without cervical lymphadenopathy or microcalcification, radiologist and surgeons were very conservative in their approach. This is also reflected by a large proportion of patients (44.9%) having less than a total thyroidectomy.

During 1970–2010, the DSS for thyroid cancer has improved by 10.2%, cumulative incidence of death due to thyroid cancer fell by 8.1%, whereas the DFS for patients treated with radical intent has remained unchanged (Table 1). This improvement is not because of an increase in the proportion of early-stage disease or smaller tumors in recent years (Table 1). Although both total thyroidectomy and adjuvant RAI treatment have been used more often during the recent time (Table 1), they did not have any significant impact on DSS (Table 3). To maintain homogeneity of data across the study period, only clinically and radiologically detectable disease relapses were considered as recurrences. Isolated hyperthyroglobulinemia was not considered as an evidence of recurrence. More aggressive treatment of early stage disease has not resulted in a significantly better disease control, as reflected by a stable disease free survival during this period. Therefore, the aggressive use of adjuvant RAI should be judiciously balanced against the treatment-related morbidities and the risk of developing second primary cancer.

On multivariate analysis, DSS was negatively influenced by nonpapillary tumor histology, male gender, advanced tumor stage, incomplete surgical resection, and older age at diagnosis (Table 3). Patient demographics, completeness of surgery, and initial cancer stage have not changed significantly in our study cohort (Table 1), however, there has been a marked change in the relative proportion of various types of thyroid cancer diagnosed over this time period. The proportion of papillary thyroid cancer has increased from 58% (1970–1980) to 85.9% (2000–2010) and the proportion of anaplastic cancers fell from 5.7% (1970–1980) to 2.1% (2001–2010). As papillary thyroid cancer has the best survival rates (96.8% at 10 years) and anaplastic thyroid cancer has the worst (7.3% survival at 10 years), the opposite trends in the relative incidence of papillary and anaplastic thyroid cancers is most likely responsible for the improved outcome of thyroid cancer (Tables 1 and 2). After controlling for other independent prognostic factors influencing DSS of thyroid cancer in a multivariate model, there was no difference in the DSS of thyroid cancer observed over the last four decades (Table 3).

A time span of four decades is both strength as well as a limitation of our study as the diagnostic criteria, staging, and treatment recommendations have evolved over time. We tried to circumvent this challenge by uniformly restaging all cancers using the 2009 AJCC/UICC Cancer Staging System for thyroid cancer. Additionally, as a part of our collaborative staging project, the pathology and treatment details of 683 (29.6%) cases were independently reviewed for accuracy and topography, and histology were recoded by WHO International Classification of Diseases for Oncology (3rd edition) codes. In view of prolonged course of most thyroid cancers, it is possible that other causes of mortality, unrelated to thyroid cancer, could result in overestimation of thyroid cancer deaths by Kaplan–Meir method. Consequently, we used competing risk regression to obtain unbiased estimation of cumulative incidence of deaths resulting from thyroid cancer which showed a significant change over the study period. A competing risk is an event the occurrence of which either precludes or changes the probability of occurrence of another event of interest. In this study deaths to second primary cancer and noncancer deaths were treated as competing risks.

To conclude, we observed a true increase in the incidence of thyroid cancer in the province of Manitoba that cannot be attributed to over diagnosis of subclinical disease alone. A 10.2% improvement in DSS of thyroid cancer over the last four decades in our population cohort was independent of early diagnosis or more aggressive treatment. The major factor contributing to this improvement in survival is the increasing proportion of papillary thyroid carcinoma diagnoses and a corresponding decrease in anaplastic thyroid carcinoma diagnoses, as outcomes for the former are usually excellent whereas outcomes for the latter are typically dismal.

## References

[b1] Canadian Cancer Society's Steering Committee on Cancer Statistics (2012). Canadian cancer statistics. 2012.

[b2] Enewold L, Zhu K, Ron E, Marrogi AJ, Stojadinovic A, Peoples GE (2009). Rising thyroid cancer incidence in the United States by demographic and tumor characteristics, 1980–2005. Cancer Epidemiol. Biomarkers Prev.

[b3] Jung KW, Park S, Kong HJ, Won YJ, Lee JY, Seo HG (2012). Cancer statistics in Korea: incidence, mortality, survival, and prevalence in 2009. Cancer Res. Treat.

[b4] Grodski S, Brown T, Sidhu S, Gill A, Robinson B, Learoyd D (2008). Increasing incidence of thyroid cancer is due to increased pathologic detection. Surgery.

[b5] Burgess JR, Tucker P (2006). Incidence trends for papillary thyroid carcinoma and their correlation with thyroid surgery and thyroid fine-needle aspirate cytology. Thyroid.

[b6] Colonna M, Guizard AV, Schvartz C, Velten M, Raverdy N, Molinie F (2007). A time trend analysis of papillary and follicular cancers as a function of tumor size: a study of data from six cancer registries in France (1983–2000). Eur. J. Cancer.

[b7] Davies L, Welch HG (2006). Increasing incidence of thyroid cancer in the United States, 1973–2002. JAMA.

[b8] Yu GP, Li JC, Branovan D, McCormick S, Schantz SP (2010). Thyroid cancer incidence and survival in the national cancer institute surveillance, epidemiology, and end results race/ethnicity groups. Thyroid.

[b9] Leenhardt L, Grosclaude P, Cherie-Challine L (2004). Increased incidence of thyroid carcinoma in France: a true epidemic or thyroid nodule management effects? Report from the French Thyroid Cancer Committee. Thyroid.

[b10] Lubina A, Cohen O, Barchana M, Liphshiz I, Vered I, Sadetzki S (2006). Time trends of incidence rates of thyroid cancer in Israel: what might explain the sharp increase. Thyroid.

[b11] Reynolds RM, Weir J, Stockton DL, Brewster DH, Sandeep TC, Strachan MW (2005). Changing trends in incidence and mortality of thyroid cancer in Scotland. Clin. Endocrinol. (Oxf).

[b12] Smailyte G, Miseikyte-Kaubriene E, Kurtinaitis J (2006). Increasing thyroid cancer incidence in Lithuania in 1978–2003. BMC Cancer.

[b13] Fahey TJ, Reeve TS, Delbridge L (1995). Increasing incidence and changing presentation of thyroid cancer over a 30-year period. Br. J. Surg.

[b14] Liu S, Semenciw R, Ugnat AM, Mao Y (2001). Increasing thyroid cancer incidence in Canada, 1970-1996: time trends and age-period-cohort effects. Br. J. Cancer.

[b15] Howlader N, Noone AM, Krapcho M, Neyman N, Aminou R, Waldron W SEER Cancer Statistics Review, 1975-2009 (Vintage 2009 Populations).

[b16] Canadian Cancer Society's Steering Committee on Cancer Statistics (2011). Canadian cancer statistics 2011.

[b17] Kent WDT, Hall SF, Isotalo PA, Houlden RL, George RL, Groome PA (2007). Increased incidence of differentiated thyroid carcinoma and detection of subclinical disease. CMAJ.

[b18] Manitoba Bureau of Statistics (2012).

[b19] http://www.statcan.gc.ca/tables-tableaux/sum-som/l01/cst01/demo62h-eng.htm.

[b20] Carling T, Carty SE, Ciarleglio MM, Cooper DS, Doherty GM, Kim LT (2012). American Thyroid Association Surgical Affairs Committee: American Thyroid Association design and feasibility of a prospective randomized controlled trial of prophylactic central lymph node dissection for papillary thyroid carcinoma. Thyroid.

[b21] Hedinger C (1988). Histological typing of thyroid tumours.

[b22] Galanti MR, Hansson L, Bergstrom R, Wolk A, Hjartåker A, Lund E (1997). Diet and the risk of papillary and follicular thyroid carcinoma: a population-based case–control study in Sweden and Norway. Cancer Causes Control.

[b23] Sont WN, Zielinski JM, Ashmore JP, Jiang H, Krewski D, Fair ME (2001). First analysis of cancer incidence and occupational radiation exposure based on the national dose registry of Canada. Am. J. Epidemiol.

[b24] Fincham SM, Ugnat AM, Hill GB, Kreiger N, Mao Y (2000). Is occupation a risk factor for thyroid cancer Canadian cancer registries epidemiology research group. J. Occup. Environ. Med.

[b25] Fraker DL (1995). Radiation exposure and other factors that predispose to human thyroid neoplasia. Surg. Clin. North Am.

[b26] Haselkorn T, Bernstein L, Preston-Martin S, Cozen W, Mack WJ (2000). Descriptive epidemiology of thyroid cancer in Los Angeles County, 1972–1995. Cancer Causes Control.

[b27] Han JM, Kim WB, Kim TY, Ryu JS, Gong G, Hong SJ (2012). Time trend in tumor size and characteristics of anaplastic thyroid carcinoma. Clin. Endocrinol. (Oxf).

[b28] Busco S, Giorgi Rossi P, Sperduti I, Pezzotti P, Buzzoni C, Pannozzo F (2013). Increased incidence of thyroid cancer in Latina, Italy: a possible role of detection of subclinical disease. Cancer Epidemiol.

[b29] D. S Cooper, G. M Doherty, Haugen BR, Kloos RT, Lee SL, Mandel SJ, Mazzaferri EL, McIver B, Pacini F, Schlumberger M, Sherman SI, Steward DL, Tuttle RM, American Thyroid Association (ATA) Guidelines Taskforce on Thyroid Nodules and Differentiated Thyroid Cancer (2009). Revised American Thyroid Association management guidelines for patients with thyroid nodules and differentiated thyroid cancer. Thyroid.

